# Supplementary material for: Predicting the protein interaction landscape of a free-living bacterium with pooled-AlphaFold3
Source: Mol Syst Biol. 2026 Jan 20;22(4):497–518. doi: 10.1038/s44320-026-00189-7 (PMC13047044; doi:10.1038/s44320-026-00189-7)
Supplement: Supplementary file 1 — Appendix [file 44320_2026_189_MOESM1_ESM.pdf]

**APPENDIX**

# Predicting the protein interaction landscape of a free-living bacterium with pooled-AlphaFold3

**AUTHORS:** Horia Todor[1,7,*], Lili M. Kim[1,6], Jürgen Jänes[5,6], Hannah N. Burkhart[1], Seth A. Darst[4], Pedro Beltrao[5], Carol A. Gross[1,2,3]

**AFFILIATIONS:**

[1]Department of Microbiology and Immunology, University of California, San Francisco, San Francisco, CA 94158, USA

[2]Department of Cell and Tissue Biology, University of California, San Francisco, San Francisco, CA 94158, USA.

[3]California Institute of Quantitative Biology, University of California, San Francisco, San Francisco 94158, CA, USA.

[4]Laboratory of Molecular Biophysics, The Rockefeller University, New York, NY, USA.

[5]Institute of Molecular Systems Biology ETH Zürich, Zürich, Switzerland.

[6]These authors contributed equally

[7]Lead Contact

*Correspondence: horia.todor@gmail.com

## Table of Contents

**Appendix Figure S1: AUROC values for different methods of correcting size bias. ............................................................................................................ 2**

Raw ipTM scores demonstrated a strong and significant correlation with the square root of the summed size of interacting proteins. This relationship allows us to calculate an “expected_ipTM” for each protein pair. There are two straightforward ways to correct ipTM scores:

1. (actual_ipTM - expected_ipTM)
2. (actual_ipTM / expected_ipTM)

In the manuscript, we use the subtraction-based approach “size-corrected ipTM” (1), because its similarity to raw ipTM makes existing intuitions broadly applicable. However, the division-based approach “ipTM-ratio” (2) actually performs better than size-corrected ipTM in predicting known interactions (Appendix Figure S1, Dataset EV4).

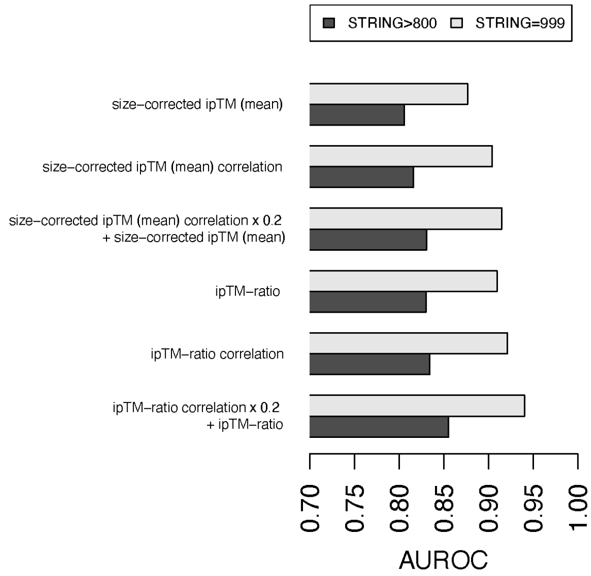


**Appendix Figure S1. AUROC values for different methods of correcting size bias.** Data is in Dataset EV4.

The success of a division-based size correction in predicting known interactions suggests that interactions involving small proteins (<200aa) are significantly undervalued by both raw ipTM and the subtraction-based size-corrected ipTM used in this manuscript. For these proteins, ipTMs within the “borderline” range of 0.05-0.2 using the subtraction-based correction may represent strong interactions, warranting close attention. ipTM-ratio values are included in Dataset EV4.
