# Supplementary material for: Predicting the protein interaction landscape of a free-living bacterium with pooled-AlphaFold3
Source: Mol Syst Biol. 2026 Jan 20;22(4):497–518. doi: 10.1038/s44320-026-00189-7 (PMC13047044; doi:10.1038/s44320-026-00189-7)
Supplement: Supplementary file 2 — Table EV1 [file 44320_2026_189_MOESM2_ESM.docx]

**Table EV1**

|  | *M. genitalium* RNAP holoenzyme^a^ | | | | | |  |  |  |  |  |  |
| --- | --- | --- | --- | --- | --- | --- | --- | --- | --- | --- | --- | --- |
| subunit | αI^b^ | αII^b^ | β | β' | δ | σ^A^ | results of PyMOL align command^c^ |  | NusG-β' | GreA-β' | Spx-α(CTD) | results of PyMOL align command |
| chain | A | B | C | D | E | F | rmsd (Å)  (# of α-carbons) |  | PDB 6C6U | PDB 6RI7 | PDB 3IHQ | rmsd (Å)  (# of α-carbons) |
|  |  |  |  |  |  |  |  |  |  |  |  |  |
| pooled AF3 job |  |  |  |  |  |  |  |  |  |  |  |  |
| allbyall5 |  | F |  | E | P |  | 0.845 (1,091) |  |  |  |  |  |
| allball98 |  |  | C | E |  |  | 0.746 (2,233) |  |  |  |  |  |
| allbyall256 | H |  |  |  |  |  | 0.372 (205) |  |  |  | L | 0.536 (84) |
| allbyall288 |  |  | A |  |  |  | 1.295 (1,116) |  |  |  |  |  |
| allbyall307 |  |  | C |  |  |  | 0.867 (1,111) |  |  |  |  |  |
| allbyall535 |  |  |  | N |  |  | 0.776 (972) |  |  |  |  |  |
| allbyall692 |  |  | E |  |  |  | 0.665 (1,019) |  |  |  |  |  |
| allbyall794 |  |  |  | C |  | D | 1.149 (1,390) |  |  |  |  |  |
| allbyall832 |  |  | E | I |  |  | 0.685 (2,138) |  |  |  |  |  |
| allbyall1130 | E |  | G |  |  |  | 0.685 (1,348) |  |  |  |  |  |
| allbyall1290 |  |  |  | L |  |  | 0.915 (1,067) |  | B |  |  | 1.479 (887) |
| allbyall1581 | A |  | F |  |  |  | 0.753 (1,312) |  |  |  |  |  |
| allbyall1604 |  |  | J |  |  |  | 0.999 (1,138) |  |  |  |  |  |
| allbyall1607 |  |  |  | I |  |  | 0.891 (1,099) |  |  | G |  | 1.577 (1,015) |
| allbyall1619 |  | J |  | E |  |  | 0.695 (1,109) |  |  |  |  |  |
| albyall2016 |  |  |  | A |  | D | 1.157 (1,473) |  |  |  |  |  |

^a^ The *M. genitalium* RNAP holoenzyme (αI-αII-β-β'‑δ-σ^A^) structure was predicted using AlphaFold3.

^b^ Calculation for αI and αII included only residues 1-250 (excluding the flexible αCTD and linker).

^c^ RMSD values were calculated using the PyMOL align command. For example, for allbyall5 (250310_mgen_allbyall_5; all pooled AF3 jobs described in Dataset EV2) against *M. genitalium* RNAP (Mge_RNAP):

align (allbyall5 and ((chain F and res 1:250) or chain E or chain P) and name CA),(Mge_RNAP and ((chain B and res 1:250) or chain D or chain E) and name CA)

Gene names for RNAP subunits: (α) RpoA/MG_177; (β’) RpoC/MG_340; (β) RpoB_MG_341; (δ) RpoE/MG_022; (σ^A^) SigA/MG_249.
