# Supplementary material for: Predicting the protein interaction landscape of a free-living bacterium with pooled-AlphaFold3
Source: Mol Syst Biol. 2026 Jan 20;22(4):497–518. doi: 10.1038/s44320-026-00189-7 (PMC13047044; doi:10.1038/s44320-026-00189-7)
Supplement: Supplementary file 11 — Expanded View Figures [file 44320_2026_189_MOESM11_ESM.pdf]

Expanded View Figures

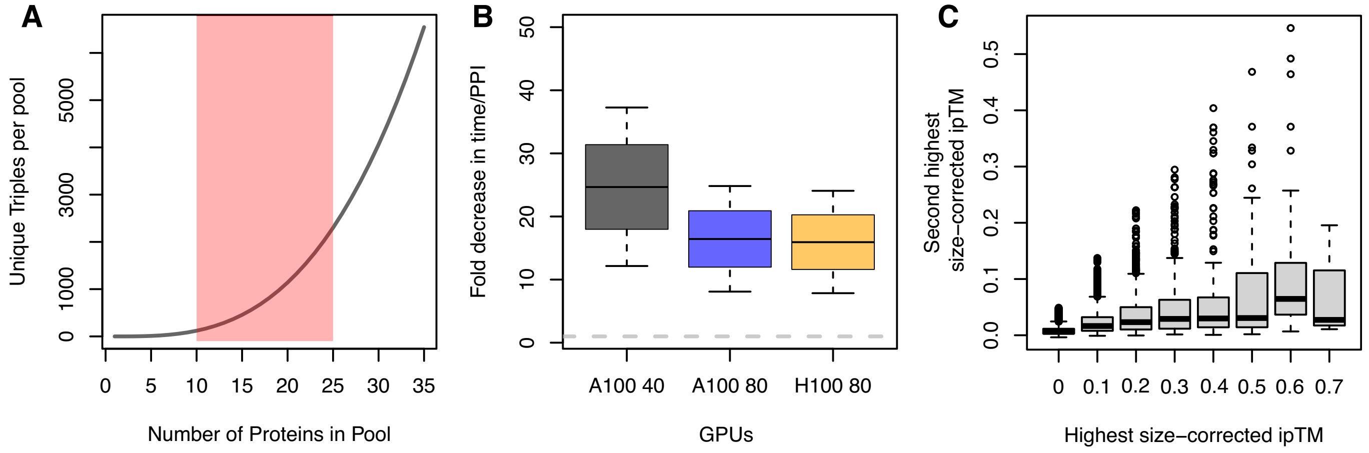

Figure EV1. Pooled-PPI prediction drastically reduces the number of required jobs and increases inference throughput for identifying tripartite interactions.

(**A**) The number of proteins in the pool determines the number of unique tripartite interactions screened in a single job. The red shaded area encompasses most jobs. (**B**) Pooled-PPI-prediction decreases inference time per unique tripartite PPI even considering the increased runtime of larger jobs. All timing estimates (1024–5120 tokens) and pool sizes of 10-25 proteins are considered and reflected in the error bars. Box bounds: first and third quartile, midline: median, whiskers: most extreme datapoints. (**C**) Tripartite interactions are rare in our dataset – most proteins in most jobs have 0 or 1 strong interactions. Even in cases where a protein strongly interacts with 2 partners, identifying a true tripartite interaction would require having additional pools with each of the two partners individually and most large complexes are accurately modeled in a pairwise manner (described later in the manuscript). Plot compares the highest size-corrected ipTM to the second highest size-corrected ipTM for all proteins in each pool ($n = 25,985$). Box bounds: first and third quartile, midline: median, whiskers: most extreme datapoints within 1.5× interquartile range.

© The Author(s)

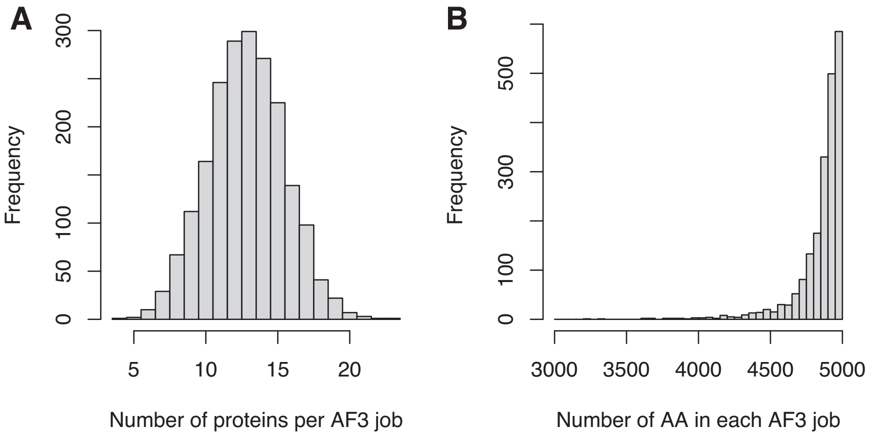

Figure EV2. Characteristics of the *M. genitalium* pools.

(**A**) Histogram of pool sizes (Datasets EV1 and EV2). (**B**) Histogram of total job size (in amino acids, Datasets EV1 and EV2).

 © The Author(s)

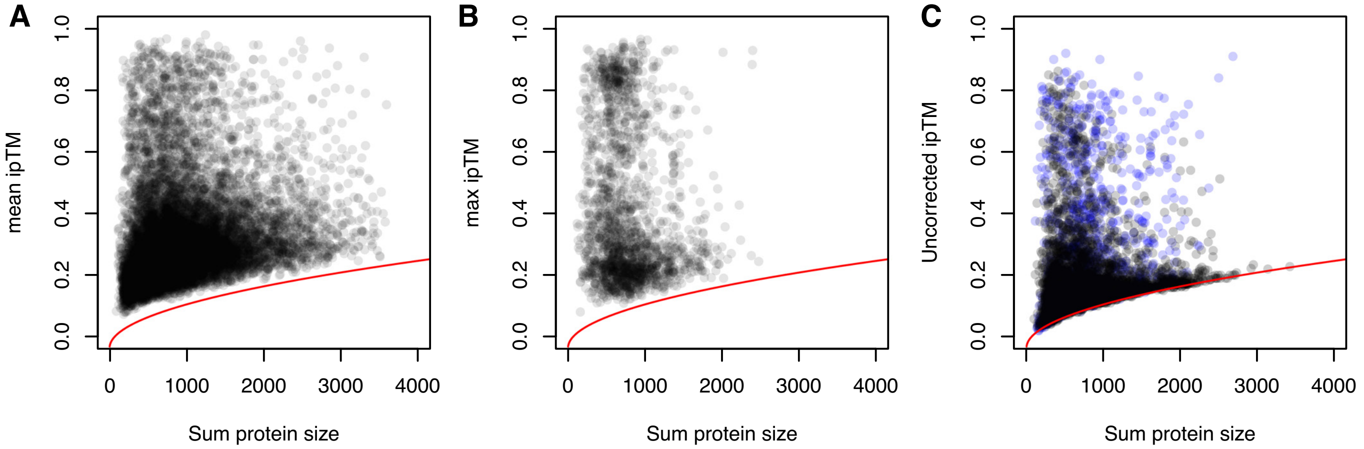

Figure EV3. Size-bias of ipTM scores is not limited to pooled approaches or to AlphaFold3.

Red line in all panels is the fit line from AlphaFold3 pools: sqrt(sum_protein_size)*0.0044–0.036. (**A**) Summed protein size plotted by ipTM for ColabFold v1.5.2/AlphaFold-Multimer from (Schmid and Walter, 2025) exhibits a similar pattern to our data, albeit a lower correlation (robust $R^2 = 0.162$, $n = 13{,}274$), likely due to a lower proportion of non-interacting protein pairs. (**B**) Summed protein size plotted by ipTM for AlphaFold-Multimer v2.1.0 data from (O'Reilly et al, 2023) exhibits a similar pattern to our data, albeit an even lower correlation (robust $R^2 = 0.021$, $n = 1977$), likely due to a very low proportion of non-interacting protein pairs. (**C**) Summed protein size plotted by ipTM for individual pairs folded by AlphaFold3 exhibits a similar pattern albeit a lower correlation. Two sets of data are shown. (1) Protein pairs selected primarily from high-scoring interactions in pools and run on alphafoldserver.com (robust $R^2 = 0.170$, $n = 942$, blue). (2) Random sample of 4560 protein pairs run using a local implementation of AlphaFold3 (robust $R^2 = 0.236$, $n = 4560$, black).

© The Author(s)

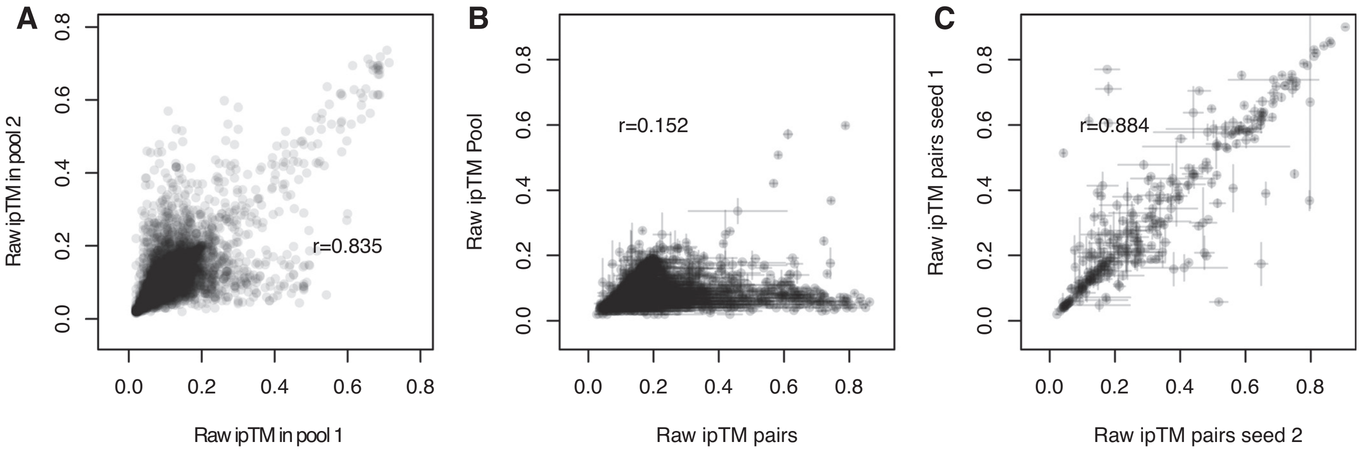

Figure EV4. ipTM-size bias camouflages variability in AlphaFold3.

(**A**) Raw ipTM scores for the 38,718 protein pairs that appear in multiple pools are similar (Pearson's $r = 0.835$, 38,718 protein pairs). (**B**) Raw ipTM is similar in paired and pooled AlphaFold3 jobs (Pearson's $r = 0.152$, 4560 pairs). Error bars represent the standard deviation of 5 diffusion samples per seed. (**C**) Raw ipTM scores across identical paired runs using different random seeds exhibit surprising variability (Pearson's $r = 0.884$, 314 pairs). Error bars represent the standard deviation of 5 diffusion samples per seed.

© The Author(s)

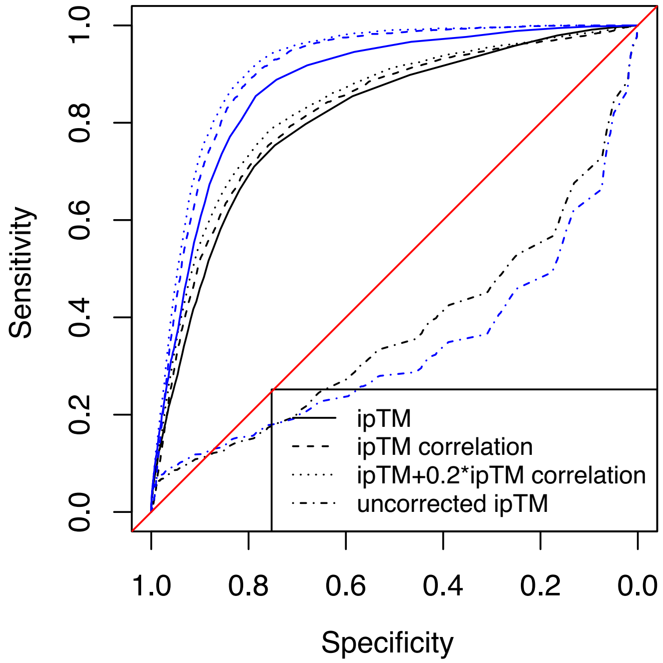

Figure EV5. Pooled-AlphaFold3 accurately predicts known interactions in the STRING database.

AUROC curve for size-corrected ipTM scores (solid line), size-corrected ipTM correlations (large dashed line), size-corrected ipTM + 0.2 size-corrected ipTM correlation (small dashed line), and uncorrected ipTM (alternating small and large dashed line). Black lines consider interactions with STRING experimental scores >800 (strong interactions) as the true-positive set. Blue lines consider interactions with STRING experimental scores = 999 (strongest interactions) as the true-positive set.

© The Author(s)

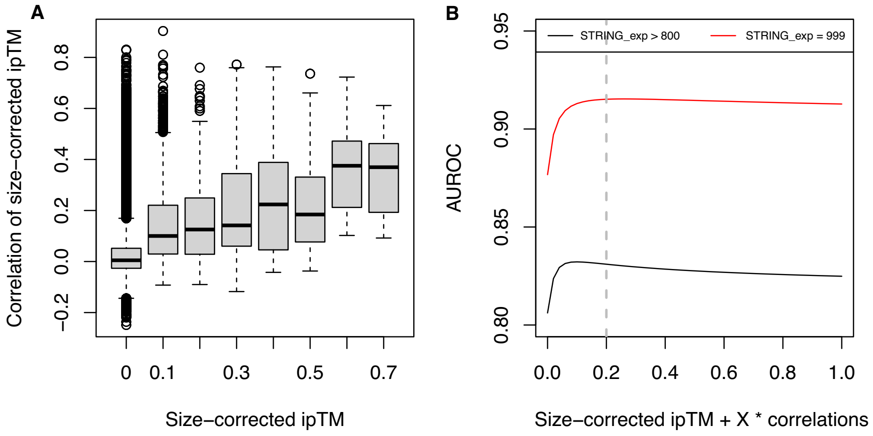

Figure EV6. Size-corrected ipTM and the correlation between the size-corrected ipTM of two proteins are partially orthogonal, and combining the two increases predictive performance.

(**A**) Boxplot showing partial correlation between the size-corrected ipTM of two proteins and the correlation between their size-corrected ipTM profiles. (**B**) AUROC benchmarking the STRING experimental dataset with different combinations of size-corrected ipTM and its correlation. For STRING > 800 (black line), the maximum AUROC is 0.832 and is achieved at (size-corrected ipTM + 0.1 correlation). For STRING = 999 (red line), the maximum AUROC is 0.915 and is achieved at (size-corrected ipTM + 0.26 correlation). We use a combined score of (size-corrected ipTM + 0.2 correlation) for the remainder of the manuscript (dashed vertical line).

 © The Author(s)

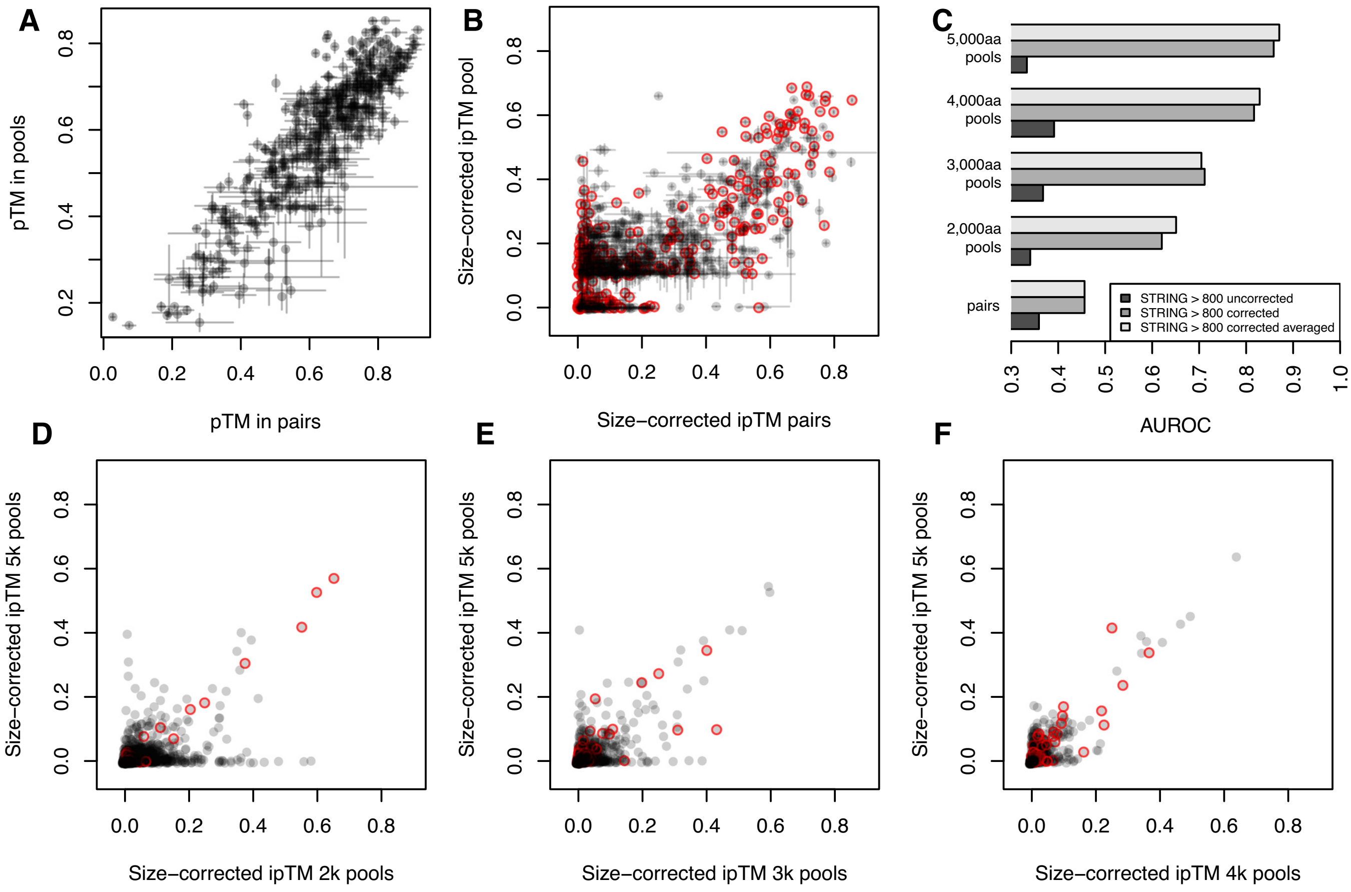

Figure EV7. Larger pool sizes are more predictive of known PPI and exhibit fewer false-positive hits.

Red circles in (**B**, **D–F**) represent protein pairs with STRING experimental scores >800. (**A**) Average pTM scores for all 418 proteins for which we had both pooled and paired data. pTMs were highly correlated between the pairs and pools (Pearson's $r = 0.882$) and did not exhibit systematic differences (median difference = 0.039), indicating that folding in pools does not affect AlphaFold3's ability to predict monomer structures. (**B**) Size-corrected ipTMs of protein pairs with high scores in the pools are well correlated. (Pearson's $r = 0.725$, 942 pairs), though ~0 to 0.2 lower in pools. (**C**) AUROC for ~4500 protein pairs assayed using randomly generated pools of different sizes. Raw, corrected, and corrected averaged data is shown. (**C**) ROC curves for the different pool sizes. (**D–F**) Comprehensive pooled ipTMs compared to ipTMs from 2000 aa (**D**), 3000 aa (**E**) and 4000 aa (**F**) pools.

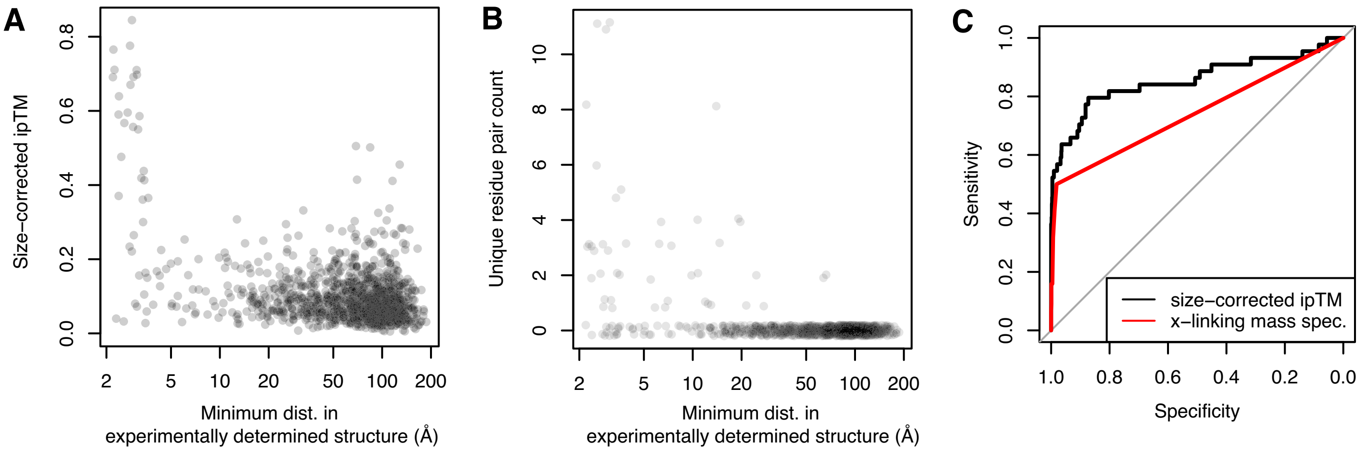

Figure EV8. Additional information about the ribosome structure prediction.

(**A**) Plot of size-corrected ipTM versus minimum distance for all ribosomal protein pairs. (**B**) Plot of number of unique cross-links versus minimum distance for all ribosomal protein pairs. Some of the additional cross-links in the XL-MS data may be due to the linker length of the cross-linking reagents. (**C**) AUROC curve showing the performance of XL-MS (red) and size-corrected ipTM for identifying ribosomal protein pairs within 5 Å.

 © The Author(s)
